# Brain Iron Deposition Alterations in Type 2 Diabetes Mellitus Patients With Mild Cognitive Impairment Based on Quantitative Susceptibility Mapping

**DOI:** 10.1111/1753-0407.70052

**Published:** 2025-01-22

**Authors:** Qiuyue Zhao, Subinuer Maimaitiaili, Yan Bi, Ming Li, Xin Li, Qian Li, Xinyi Shen, Min Wu, Linqing Fu, Zhengyang Zhu, Xin Zhang, Jiu Chen, Anning Hu, Zhou Zhang, Wen Zhang, Bing Zhang

**Affiliations:** ^1^ Department of Radiology, Nanjing Drum Tower Hospital, Affiliated Hospital of Medical School Nanjing University Nanjing City, Jiangsu Province China; ^2^ Institute of Medical Imaging and Artificial Intelligence Nanjing University Nanjing City, Jiangsu Province China; ^3^ Department of Vascular Surgery, Nanjing Drum Tower Hospital, Affiliated Hospital of Medical School Nanjing University Nanjing City, Jiangsu Province China; ^4^ Department of Endocrinology, Nanjing Drum Tower Hospital, Affiliated Hospital of Medical School Nanjing University Nanjing China

**Keywords:** iron deposition, magnetic resonance imaging, mild cognitive impairment, quantitative susceptibility mapping, type 2 diabetes mellitus

## Abstract

**Background:**

Iron is one of the most important elements in brain that may has a direct impact on the stability of central nervous system. The current study devoted to explore the alterations of iron distribution across the whole brain in type 2 diabetes mellitus (T2DM) patients with mild cognitive impairment (MCI).

**Methods:**

The quantitative susceptibility mapping (QSM) technique was used to quantify the intracranial iron content of 74 T2DM patients with MCI and 86 T2DM patients with normal cognition (NC). The group comparison was performed by a voxel‐based analysis. Then we evaluated the relationships between cognitive indicators and magnetic susceptibility value (MSV) measured by QSM of the significant brain areas, which were set as the regions of interest (ROIs). In addition, we analyzed the moderation effects of grey matter volume (GMV) of the related brain areas and several metabolic and cerebrovascular factors on the associations between MSV of ROIs and cognitive characteristics.

**Results:**

T2DM patients with MCI exhibited a lower MSV in the right middle temporal gyrus (MTG) compared to NC group. And in the MCI group, there were significantly negative correlations between MSV of the right MTG and several memory indexes. Furthermore, the moderation effects of GMV of the whole brain and the bilateral MTG on the relationship between MSV of the right MTG and scores of list recognition were significant.

**Conclusions:**

T2DM patients with MCI had a temporary decreased iron content in the right MTG, which may partially compensate for cognitive impairment.

**Trial Registration:** The study was registered at Clinicaltrials.gov (NCT02738671)

## Graphical abstract

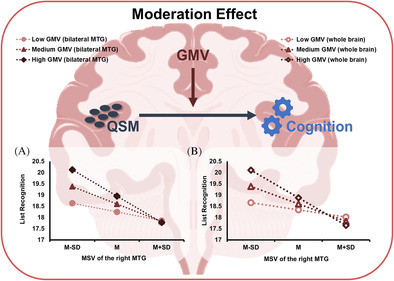

**Summary**

- A lower iron content in the right middle temporal gyrus (MTG) was found among the type 2 diabetes mellitus (T2DM) patients with mild cognitive impairment (MCI) compared to T2DM patients with normal cognition.
- Significant negative correlations existed between iron burden of the right MTG and memory indices in the MCI group.
- The moderation effects of grey matter volume of the related brain areas on the relationship between iron content of the right MTG and memory index were significant in the MCI group.

## 1 Introduction

Type 2 diabetes mellitus (T2DM) is a pervasive metabolic disease worldwide, which severely reduces the quality of life and life expectancy of patients [1]. In addition to the well‐acknowledged disorder in blood glucose metabolism associated with T2DM, an expanding body of research has elucidated its direct effect on patients' cognitive function [2, 3]. Compared with age‐matched healthy controls, T2DM patients exhibit cognitive impairment, notably in domains encompassing memory, executive function and processing speed [4]. Therefore, an exploration in the neuropathological mechanisms underlying cognitive impairment in patients suffering from T2DM deserves more attention.

Iron is a vital chemical element in brain that contributes to many essential biological processes in a homeostatic state, including oxygen transport, DNA synthesis, neurotransmitter synthesis and metabolism [5, 6, 7]. However, excessive accumulation of iron within brain could lead to oxidative damage and neuronal apoptosis, which are well‐documented contributors to various neurodegenerative diseases [8, 9]. Insulin resistance plays a vital role in the pathogenesis of T2DM [10]. It is characterized by impaired insulin utilization that necessitating higher‐than‐normal insulin dose for regulating blood glucose levels [11]. Prior researches illuminated a strong correlation between insulin resistance and perturbed cerebral iron homeostasis [12, 13]. Accordingly, many neuroimaging studies focused on measuring brain iron metabolism of T2DM patients and observed the disordered iron deposition in regional brain areas, while exhibited the link between disrupted iron homeostasis in brain and impaired cognition in patients with T2DM [14, 15]. These studies reminded us that an in‐depth investigation of iron load in central nervous system (CNS) may contribute to understand the neural mechanisms of cognitive impairment in T2DM patients.

Quantitative susceptibility mapping (QSM) is a rising magnetic resonance imaging (MRI) technique utilized to quantify the tissue magnetic susceptibility [16, 17]. There is a substantial correlation between magnetic susceptibility value (MSV) calculated by QSM and the tissue iron content [18, 19]. Consequently, QSM has been widely used in recent neuroimaging studies aimed at quantifying cerebral iron content [20, 21, 22]. Previous investigations of aberrant patterns in brain iron deposition in neurodegenerative conditions have mainly focused on subcortical grey matter nuclei, in which have disproportionately high concentration of iron [23, 24]. However, the iron sensitivity in cortical regions improves with age and limited regions of interest (ROIs) sampling likely restricted the detection of abnormalities in other brain regions [25]. Consequently, where technology permitted, we made a voxel‐based comparison of iron load in whole grey matter, which enabled the identification of novel patterns of whole‐brain iron distribution in T2DM‐MCI patients [21, 26, 27].

In this study, we measured the brain iron content through QSM and compared the difference in intracranial iron burden between T2DM patients with mild cognitive impairment (MCI) and those with normal cognition (NC) by a voxel‐based analysis. Then the correlations between MSV of ROIs and behavioral data were analyzed in each group. Grey matter volume (GMV), a fundamental neuroimaging parameter, has been extensively investigated in patients with T2DM and was closely associated with individual behavioral performance [28, 29]. Iron metabolism in brain may contribute to grey matter atrophy by facilitating the production of reactive oxygen and toxic free‐radicals, triggering neuronal cell and astrocyte death. And the mechanism has been implicated in neurodegenerative diseases [30]. Recent studies have further reinforced this connection by demonstrating significant associations between regional magnetic susceptibility and GMV, underscoring the potential role of iron metabolism in shaping grey matter morphology [31, 32]. These well‐established links between brain iron, grey matter morphology and cognitive decline formed a moderation effect, by which we assessed whether GMV in specific brain areas could influence the relationship between brain iron content and cognitive performance. Besides, we expanded our moderation analyses to include metabolic (glycated hemoglobin A1c (HbA1c), fasting blood glucose (FBG), postprandial blood glucose (PBG) and homeostasis model assessment of insulin resistance (HOMA‐IR)) and cerebrovascular factors (the volume of white matter hyperintensity (WMH)) strongly associated with T2DM‐related neuropathology and cognitive decline as potential moderators. In conclusion, the current study devoted to detect the brain‐wide distribution patterns of iron in T2DM patients with MCI and its correlations with cognitive impairment.

## 2 Methods

### 2.1 Participants

One hundred and sixty‐two patients with T2DM were recruited from the endocrinology department of the Nanjing Drum Tower Hospital, affiliated hospital of Medical School, Nanjing University. The diagnosis criteria of T2DM were according to the standards of medical care in diabetes from the American Diabetes Association. The exclusion criterias included: (1) mental or neurologic disorders that may impair cognitive abilities; (2) a family history of dementia; (3) cerebrovascular disease confirmed with MRI; (4) a history of alcohol dependency or substance abuse; (6) an experience of diabetic ketoacidosis and frequent hypoglycemia during the past two years; (7) thyroid dysfunction, anaemia or steroid treatment (8) contraindications for MRI scan.

The protocol of this study was approved by the Ethics Committee of Nanjing Drum Tower Hospital, in accordance with the Helsinki Declaration and registered at Clinicaltrials.gov (NCT02738671). And the ethics committee approved the protocol in January 2016. All participants provided written informed consent after a detailed introduction of the study process according to institutional guidelines.

### 2.2 Clinical Information and Biochemical Parameters

Fasting blood sample of each participant was collected for laboratory examination data, including FBG, HbA1c, triglycerides (TG), total cholesterol (TC), high‐density lipoprotein (HDL) and low‐density lipoprotein (LDL). Body matter index (BMI) represented as weight in kilograms divided by the square of height in meters was also calculated for each participant. Blood pressure was measured in the morning. Hypertension was identified as systolic blood pressure > = 140 mmHg or diastolic blood pressure > = 90 mmHg or taking antihypertensive medications.

### 2.3 Neuropsychological Tests

The Montreal Cognitive Assessment (MoCA) was administered to assess the global cognition [33]. A cut‐off of 26 was set to distinguish MCI cases from NC cases in the T2DM patients, with a total score of 25 or less indicating cognitive impairment [34]. Moreover, the Repeatable Battery for the Assessment of Neuropsychological Status (RBANS) was used to assess various cognitive functions of each participant, including immediate and delayed memory, visuospatial/constructional skills, attention and language [35, 36].

### 2.4 Image Data Acquisition

Image data were collected by using a 3.0‐T MR imaging scanner (Achieva TX; Philips, Netherlands) with a standard 8‐channel head coil. The SWI images were collected using a 3D multi‐echo gradient‐echo sequence with the following parameters: repetition time (TR)/echo time (TE) = 51/7.0 ms; field of view (FOV) = 216 × 216 mm and voxel size = 0.75 × 0.75 × 2 mm. A high‐resolution brain volume sequence was used to obtain individual T1‐weighted image: TR/TE = 9.7/4.6 ms; flip angle (FA) = 8^o^; FOV = 256 × 256 mm and slice thickness = 1 mm, voxel size = 1 × 1 × 1 mm. To calculate the volume of WMH, individual T2‐FLAIR image was acquired using the following scanning parameters: TR/TE = 11000/100 ms; inversion time = 2800 ms; FA = 110^o^; matrix size = 384 × 384; voxel size = 0.6 × 0.6 × 5 mm and slice thickness = 5 mm.

### 2.5 Imaging Preprocessing

#### 2.5.1 QSM Preprocessing and Quantitative Analysis

According to the published methods [37], the brain QSM maps were acquired by combining phase preprocessing and susceptibility map estimation. First, preprocess the unwrapped phase by extracting the Laplacian boundary value and then filter variable spherical mean‐value with a kernel of 8 mm radius. Second, the Multi‐Scale Dipole Inversion algorithm was used to estimate susceptibility maps [38]. QSM spatial normalization was performed using QSMexplorer (https://gitlab.com/acostaj/QSMexplorer). The QSM template was created based on normalization tools ANTs (http://stnava.githib.io/ANTs).

#### 2.5.2 Voxel‐Based Morphometry

A voxel‐based morphology analysis was conducted by using the CAT12 toolbox (http://dbm.neuro.uni‐jena.de/cat). The GMV maps of all participants were acquired after preprocessing, which included segmentation, normalizing to the standard Montreal Neurological Institute (MNI) template, resampling into a cubic voxel of 1.5 mm, tissue probability modulation, quality check and smoothing with a Gaussian kernel of 8 mm full width at half‐maximum.

#### 2.5.3 WMH Preprocessing and Quantitative Analysis

To evaluate the WMH, brain MRI of participants were processed by use of uAI Research Portal, a one‐stop image analysis platform [39]. Then the segementation and quantification of WMH based on MR images were performed using the 2D VB‐Net algorithm [40]. And the WMH were divided into 4 categories on the basis of their distance from the ventricle, including juxtaventricular WMH (JVWMH), an area within 3 mm from the ventricle; periventricular WMH (PWMH), an area 3–13 mm away from the ventricle; juxtacortical WMH (JCWMH), an area within 4 mm from the corticomedullary; and deep WMH (DWMH), an area between PWMH and JCWMH [41].

### 2.6 Statistical Analysis

#### 2.6.1 Demographic, Clinical and Behavioral Data

The enrolled T2DM patients were divided into T2DM‐MCI and T2DM‐NC groups according to their scores of MoCA. Intergroup differences in demographic, clinical and cognitive data were evaluated by SPSS 25.0. The Chi‐square test was used for categorical variables, and the two‐sample *t*‐test was used for continuous variables. The significant level was set at *p* < 0.05.

#### 2.6.2 Neuroimaging Data

A voxel‐wise two‐sample *t*‐test was executed to determine the differences in MSV of whole grey matter between the two groups. Age, gender, education years and BMI were treated as confounding variables. A priori grey matter (GM) template binarized with a threshold > 0.5 was used as a mask to confine the statistical analysis within the GM regions. Gaussian random field (GRF) in DPABI software was applied to multiple comparison analysis with the following parameters: voxel‐level *p* < 0.001, cluster‐level *p* < 0.05 and estimated smoothing kernel. The brain regions corrected by multiple comparison were established as the ROIs, where MSV were extracted to perform the partial correlation analyses with cognitive parameters after controlling age, gender, education years and BMI in two groups, respectively. Bonferroni‐corrected *p* < 0.05 was considered statistically significant.

**FIGURE 1:**
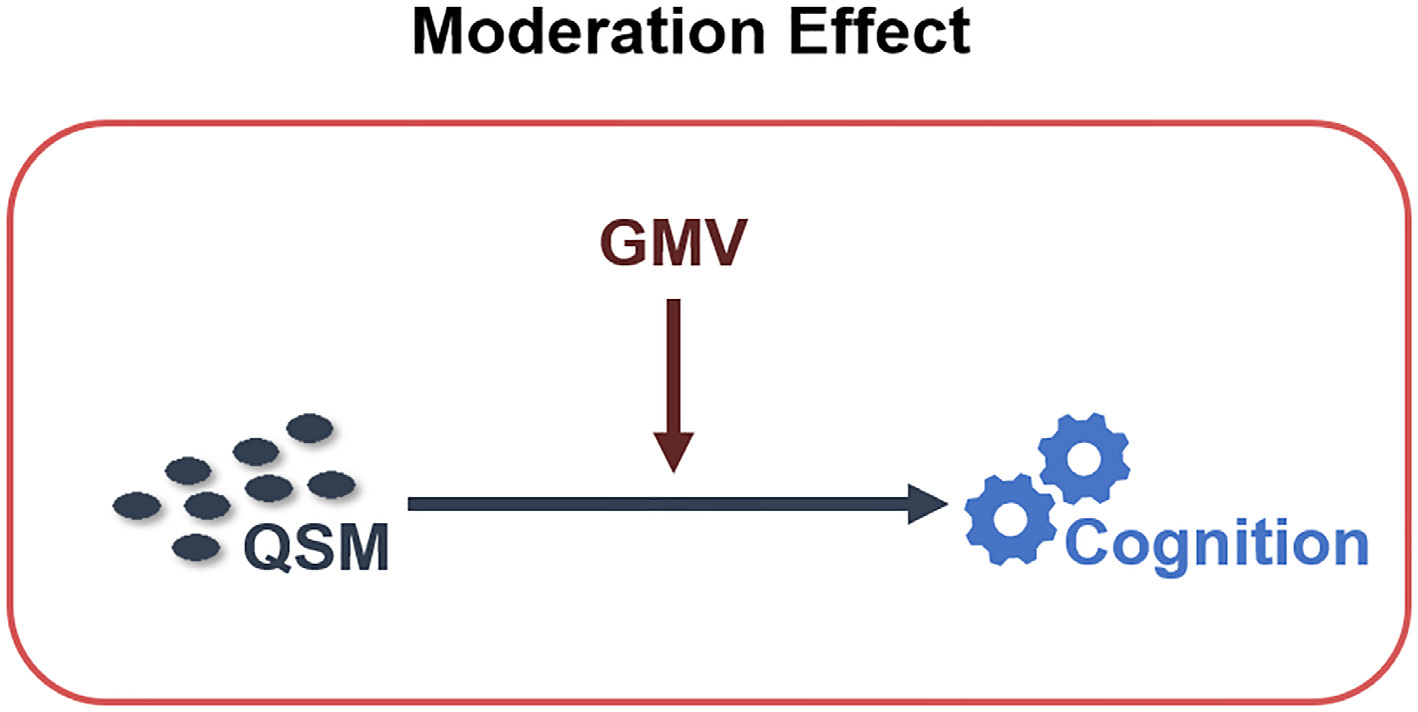
The moderation effect model of GMV of the related brain areas on the associations between MSV of ROIs and cognitive indexes. GMV, grey matter volume; MSV, magnetic susceptibility value; QSM, quantitative susceptibility mapping; ROIs, regions of interest. (Own data, 2016–2019).

Based on the results of the partial correlation analyses above, GMV of the whole brain and brain areas where ROIs are located were extracted as moderators to perform the moderation effect analyses between MSV of ROIs and cognitive indexes by using PROCESS (http://www.processmacro.org/index.html) plugged in the SPSS 25.0. The moderation effect model is shown in Figure 1. We defined MSV of ROIs as the independent variables, GMV indexes as the moderator variables and cognitive parameters as the dependent variables. As well, age, gender, education years and BMI were considered nuisance variables. Other parameters in the model were as following: model number = 1, bootstrap samples number = 5000 and confidence interval (CI) = 95%. When the 95% CI of the interaction effect of independent and moderator variables does not contain zero, the moderation effect is considered statistically significant. In addition, we included several important metabolic and cerebrovascular factors as the potential moderators in the brain iron‐cognitive level relation, employing the same methodological framework as described above.

## 3 Results

### 3.1 Demographic, Clinical and Behavioral Data

After excluding two paritcipants failing to complete the MRI scan, 160 T2DM patients were ultimately retained in the study, of which 74 suffered from MCI. Details of demographic and clinical characteristics of the 160 participants are listed in Table 1. Descriptions of the cognitive parameters are shown in Table 2. There were no significant difference in age, gender and education years between the T2DM‐MCI group and the T2DM‐NC group (*p* > 0.05). Compared to the NC group, the MCI group scored lower in MoCA and most of the subtests of RBANS (*p* < 0.05).

**TABLE 1:** Demographic and clinical characteristics of study participants.

Characteristics	T2DM‐MCI group (*n* = 74)	T2DM‐NC group (*n* = 86)	*p*
Demographics			
Age (years)	60.27 ± 5.66	58.50 ± 7.78	0.107
Male (number (%))	44 (59)	58 (67)	0.295**
Education (years)	11.62 ± 2.82	12.62 ± 3.53	0.052
Clinical data			
BMI (kg/m^2^)	23.66 ± 2.42	25.03 ± 3.09	0.002*
Hypertension (number (%))	34 (46)	38 (44)	0.823**
TG (mmol/L)	1.48 ± 0.87	1.57 ± 0.90	0.517
TC (mmol/L)	4.55 ± 0.98	4.70 ± 1.33	0.416
LDL	2.64 ± 0.85	2.76 ± 1.13	0.448
HDL	1.29 ± 0.27	1.27 ± 0.37	0.686
FBG (mmol/L)	8.00 ± 2.35	8.33 ± 2.95	0.441
HbA1c (%)	8.50 ± 1.73	8.43 ± 2.02	0.820
PBG	15.80 ± 4.23	15.66 ± 4.63	0.834
HOMA‐IR	1.53 ± 0.75	1.51 ± 0.84	0.897
Course of disease	10.59 ± 7.37	10.15 ± 7.53	0.707

*Note:* Data is represented as mean ± standard deviation for continuous variables, and the categorical variables (gender and hypertension) are expressed as number (percentage).

Abbreviations: BMI, body mass index; FBG, fasting blood glucose; HbA1c, glycated hemoglobin A1c; HDL, high‐density lipoprotein; HOMA‐IR, homeostasis model assessment of insulin resistance; LDL, low‐density lipoprotein; MCI, mild cognitive impairment; NC, normal cognition; PBG, postprandial blood glucose; TC, total cholesterol; TG, triacylglycerol; T2DM, type 2 diabetes mellitus.

* *p* < 0.05 was considered significant.

** The *p* value was obtained by *χ^2^* test.

**TABLE 2:** Cognitive parameters of study participants.

Cognitive parameters	T2DM‐MCI group (*n* = 74)	T2DM‐NC group (*n* = 86)	*p*
MoCA	23.89 ± 1.30	27.48 ± 1.22	< 0.001*
RBANS indexes			
Immediate memory	71.14 ± 13.54	88.01 ± 13.60	< 0.001*
Delayed memory	87.55 ± 14.15	97.17 ± 10.75	< 0.001*
Visuospatial/construction	103.12 ± 14.23	109.03 ± 12.86	0.006*
Language	97.18 ± 7.43	99.57 ± 11.98	0.139
Attention	100.97 ± 10.09	106.41 ± 11.74	0.002*
RBANS subtests			
List learning	21.55 ± 7.62	25.42 ± 4.19	< 0.001*
Story memory	10.38 ± 3.94	13.95 ± 4.03	< 0.001*
Figure copy	18.93 ± 1.48	19.09 ± 1.43	0.484
Line orientation	16.09 ± 2.44	17.34 ± 2.44	0.002*
Picture naming	9.85 ± 0.46	9.98 ± 0.93	0.294
Semantic fluency	19.66 ± 3.72	21.38 ± 4.09	0.007*
Digit span	14.36 ± 1.82	15.22 ± 4.42	0.121
Coding	32.16 ± 9.29	39.15 ± 12.98	< 0.001*
List recall	3.66 ± 2.08	5.75 ± 2.02	< 0.001*
List recognition	18.59 ± 1.54	19.07 ± 1.30	0.036*
Story recall	5.05 ± 2.37	7.07 ± 2.23	< 0.001*
Figure recall	13.26 ± 4.00	15.12 ± 3.85	0.003*

*Note:* Data is represented as mean ± standard deviation.

Abbreviations: MCI, mild cognitive impairment; MoCA, montreal cognitive assessment; NC, normal cognition; RBANS, repeatable battery for the assessment of neuropsychological status; T2DM, type 2 diabetes mellitus.

* *p* < 0.05 was considered significant.

### 3.2 Comparison of MSV of the Whole Brain Between Groups

**FIGURE 2:**
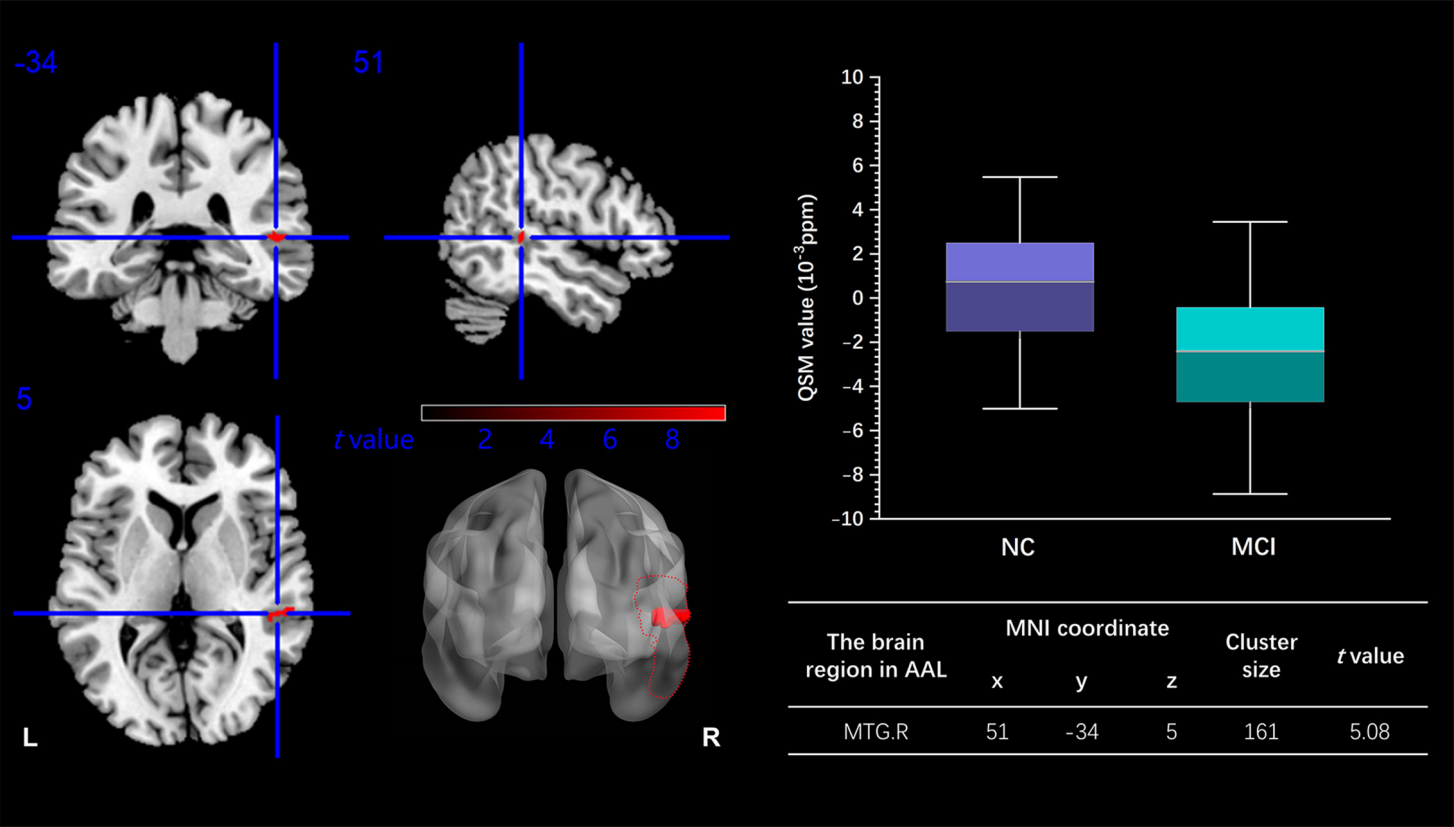
Comparison of brain‐wide MSV between the T2DM‐MCI group and T2DM‐NC group. The T2DM‐MCI group showed a significantly lower MSV in the right MTG than the T2DM‐NC group. AAL, automated anatomical labeling; L, left; MCI, mild cognitive impairment; MNI, montreal neurological institute; MSV, magnetic susceptibility value; MTG, middle temporal gyrus; NC, normal cognition; QSM, quantitative susceptibility mapping; R, right; T2DM, type 2 diabetes mellitus. (Own data, 2016–2019).

Through the voxel‐wise analysis, the T2DM‐MCI group exhibited a lower MSV in the right middle temporal gyrus (MTG) than the T2DM‐NC group (peak MNI coordinate: *x* = 51, *y* = −34, *z* = 5, cluster size = 161 voxels, GRF correction *p* < 0.05) (Figure 2).

### 3.3 Correlations Between MSV of ROIs and Cognitive Indexes in RBANS

**FIGURE 3:**
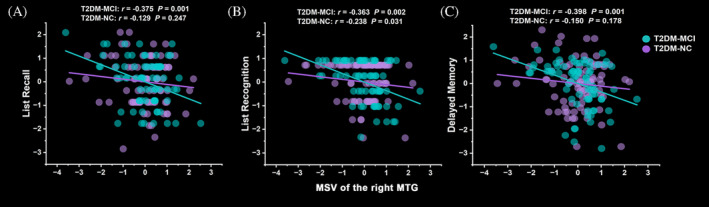
Correlations between MSV of the right MTG and scores of list recall (A), list recognition (B) and delayed memory (C) in RBANS in the T2DM‐MCI and T2DM‐NC group. The green markers represented the T2DM‐MCI group and the purple markers represented the T2DM‐NC group. MCI, mild cognitive impairment; MSV, magnetic susceptibility value; MTG, middle temporal gyrus; NC, normal cognition; RBANS, repeatable battery for the assessment of neuropsychological status; T2DM, type 2 diabetes mellitus. (Own data, 2016–2019).

In the T2DM‐MCI group, the scores of list recall (*r* = −0.375, *p* = 0.001), list recognition (*r* = −0.363, *p* = 0.002) and delayed memory (*r* = −0.398, *p* = 0.001) in RBANS were negatively correlated with MSV of the right MTG after Bonferroni correction. However, the correlations above in the T2DM‐NC group failed to pass Bonferroni correction (*p* > 0.05) (Figure 3).

### 3.4 Moderation Effects of GMV of the Related Brain Areas on the Associations Between MSV of ROIs and Cognitive Indexes

**FIGURE 4:**
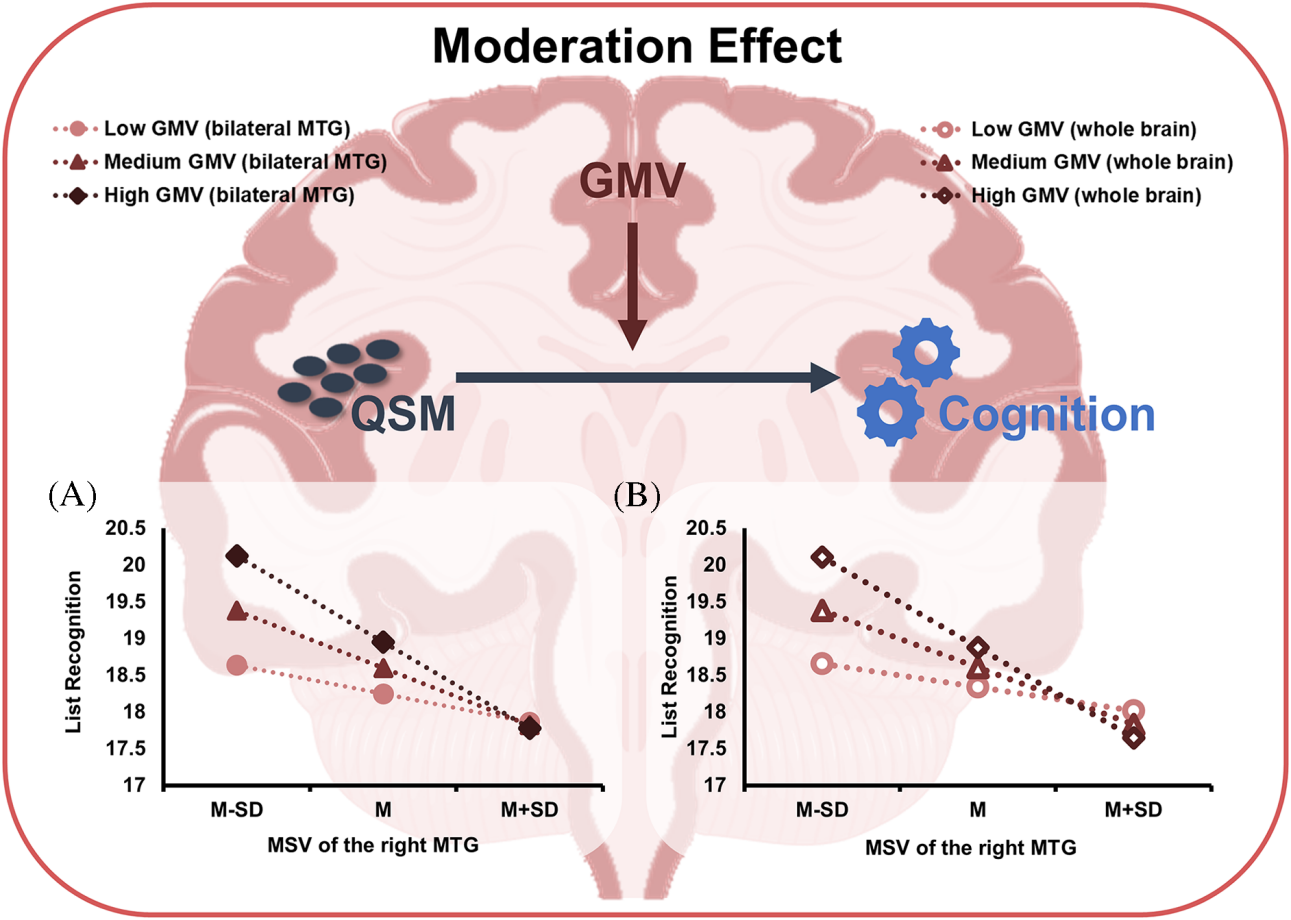
The conditional effects of levels (low, medium and high) of GMV of the bilateral MTG (A) and the whole brain (B) on the correlations between MSV of the right MTG and list recognition in the T2DM‐MCI group. GMV, grey matter volume; M, medium; M‐SD, one standard deviation below the medium; M + SD, one standard deviation above the medium; MCI, mild cognitive impairment; MSV, magnetic susceptibility value; MTG, middle temporal gyrus; QSM, quantitative susceptibility mapping; T2DM, type 2 diabetes mellitus. (Own data, 2016–2019).

GMV of the whole brain and the bilateral MTG were extracted as moderators to perform the moderation effect analyses between MSV of ROIs and cognitive parameters in the T2DM‐MCI group. The GMV above had significant positive moderation effects on the relationship between MSV of the right MTG and list recognition in RBANS (*p* < 0.05) (Table 3). In addition, the moderation effects of GMV were examined at low (one standard deviation below the mean), mean and high (one standard deviation above the mean) level, respectively. MSV of the right MTG had a stonger correlation with list recognition when GMV of the whole brain (effect size = −1.23, *p* = 0.001, 95% CI [−1.95, −0.51]) and the bilateral MTG (effect size = −1.17, *p* < 0.001, 95% CI [−1.83, −0.51]) were high and average when moderators at medium level. However, the association between MSV of the right MTG and list recognition was not significant at the low level of GMV of the whole brain (effect size = −0.32, *p* = 0.148, 95% CI [−0.76, 0.12]) and the bilateral MTG (effect size = −0.39, *p* = 0.06, 95% CI [−0.79, 0.02]). The conditional effects of predictors (MSV of the right MTG) at each level of moderators (GMV of the whole brain or the bilateral MTG) are depicted in Figure 4.

**TABLE 3:** The moderation effect of GMV in relevant brain areas on the association between MSV of the right MTG and list recognition in T2DM‐MCI group.

	Effect size	*p*	95% CI
GMV of the whole brain			
MSV of the right MTG	−0.78	< 0.001*	[−1.18, −0.38]
GMV of the whole brain	0.27	0.25	[−0.19, 0.73]
Interaction	−0.45	0.044*	[−0.90, −0.01]
GMV of the bilateral MTG			
MSV of the right MTG	−0.78	< 0.001*	[−1.17, −0.39]
GMV of the bilateral MTG	0.35	0.093	[−0.06, 0.77]
Interaction	−0.39	0.043*	[−0.77, −0.11]

Abbreviations: CI, confidence interval; GMV, grey matter volume; MCI, mild cognitive impairment; MSV, magnetic susceptibility value; MTG, middle temporal gyrus; T2DM, type 2 diabetes mellitus.

* *p* < 0.05 was considered significant.

### 3.5 Moderation Effects of Metabolic and Cerebrovascular Factors on the Associations Between MSV of ROIs and Cognitive Indexes

None of the selected metabolic and cerebrovascular factors was the significant moderators on the relationships between MSV of ROIs and cognitive indices in the T2DM‐MCI group. The statistical results are shown in the Data S1.

## 4 Discussion

In the current study, we compared the differences in whole‐brain iron deposition between the T2DM patients with MCI and those with NC based on QSM technique. We also investigated the correlations between MSV of the significant brain area and cognitive functions in each group. Furthermore, the moderation effects of GMV parameters, metabolic and cerebrovascular factors strongly related to T2DM on the associations between MSV of ROI and cognitive characteristics were explored. We found a lower MSV in the right MTG in the MCI group than in the NC group. In the T2DM‐MCI group, there were negative correlations between MSV of the right MTG and several memory indexes. And the correlation between MSV of the right MTG and list recognition was moderated by GMV of the whole brain and the bilateral MTG.

QSM is a novel MRI technique known for its noninvasive and sensitive ability in quantifying iron content of tissues and has been widely used in clinical researches [42, 43, 44]. The accuracy of QSM technique has been proved in several post‐mortem studies, which determined the significant correlations between QSM contrast and histochemical measurements of iron in grey matter [19, 45, 46, 47]. Iron is a vital cofactor involved in many important neurobiological processes, such as oxygen transportation, DNA synthesis and neurotransmitters metabolism [30]. However, brain iron overload facilitates the production of reactive oxygen species and toxic free radicals, triggering injury of nucleic acids, proteins and lipid membranes and ultimately contributing to ferrpptosis [48]. Accordingly, a growing number of studies focused on the influence of brain iron homeostasis on cognitive functions and provided good evidence to demonstrated the strong connections between over‐increased iron accumulation in brain and several typical neurodegenerative diseases [49, 50, 51].

Currently, we found that the T2DM‐MCI group had lower iron content in the right MTG compared to the T2DM‐NC group. The MTG region is particularly vulnerable to T2DM. Neuroimaging studies have consistently shown structural and functional abnormalities in the MTG in T2DM patients, particularly in those with cognitive impairment [29, 52]. Functionality, the MTG is one of the important brain areas of information convergence within CNS and plays an essential role in maintaining memory function [53, 54, 55]. Limotai [56] demonstrated that patients with temporal lobe epilepsy exhibited significant activation in the right MTG during delayed memory test. And the glucose metabolic level in the right MTG was positively correlated with the ability of delayed memory [57]. List recall and list recognition were subtests of RBANS that were used to assess personal memory functions [58]. In our study, MSV of the right MTG was negatively correlated with the above memory indices, which enhanced the responsibility of the MTG region in preserving memory functions. Meanwhile, the negative association between iron load and memory level, meaning that memory ability improved as the decrease of brain iron load, was consistent with the well‐established relationship between elevated brain iron load and neuronal damage [59, 60]. Most previous work observed increase in brain iron accumulation in neurodegenerative conditions, which are contrary to our findings [61, 62]. The dynamic characteristics of iron accumulation in brain may make the different results easy to understand. First, early histological work have shown that iron content increases with age in various brain regions, albeit with different growth patterns [63]. Therefore, age‐specific effects on brain iron accumulation and the relatively younger cohort in our study may explain the discrepancies between our findings and those from studies on age‐associated neurodegenerative diseases, such as Alzheimer's disease (AD) and Parkinson's disease (PD) [61, 62, 64]. Second, brain iron deposition is closely associated with the severity of cognitive decline and dementia. Kirsch et al. [65] and Ding et al. [66] reported significant correlations between regional iron content and both disease duration and dementia severity. Thus, the milder cognitive impairment of our participants, compared to those with AD or PD in previous studies, likely contributes to the observed differences [61, 62]. Neuropathological processes involving abnormal iron accumulation in the brain, such as oxidative stress and inflammation, are well‐documented contributors to cognitive impairment and neurodegenerative diseases [59, 60]. In our study, despite scoring lower on behavioral assessments than the NC group, patients with MCI demonstrated relatively preserved cognitive function and did not exhibit overt clinical symptoms of dementia. Based on this observation, we hypothesized that the locally decreased iron load in T2DM patients with MCI may represent a compensatory mechanism. This mechanism could aim to protect against or delay the progression to dementia in early‐stage T2DM‐MCI patients.

Besides, the significant brain area in our findings was the right MTG, rather than the subcortical nuclei typically implicated in neurodegenerative diseases. Two key factors may account for this difference. First, the use of a voxel‐based statistical method, rather than ROI‐based approaches, allowed for a broader assessment of brain iron distribution. Previous studies often focused on iron‐rich basal ganglia regions due to methodological constraints and regional iron distribution [61, 67]. Second, MCI patients in our study demonstrated significant memory deficits, supporting the susceptibility of the MTG to T2DM and its central role in memory maintenance. Furthermore, there are known anatomical and functional connections between the temporal lobe and the basal ganglia [68, 69]. Given the dynamic nature of brain iron accumulation, influenced by aging and cognitive decline, it is plausible that aberrant iron content in the right MTG contributes to iron heterogeneity in the basal ganglia as T2DM‐MCI patients aging or cognitive deterioration. A longitudinal follow‐up study is warranted to validate this hypothesis.

The moderation analyses revealed that GMV of the whole brain and the bilateral MTG were positive moderators on the relationship between MSV of the right MTG and list recognition in the T2DM‐MCI group. Moreover, the conditional analyses suggested that the moderation effects were more significant when the moderators were at a high level, that is, the negative correlation between MSV of the right MTG and list recognition was strong when GMV above were high. The stronger negative associations as GMV increasing may imply that increased GMV could strengthen the beneficial role of decreased iron load in maintaining memory levels, that is facilitating the compensatory mechanism we proposed above. As well, it is necessary for us to construct a longitudinal experiment to verify this interpretation.

There were some limitations necessary to be mentioned. First, the samples in each group were relatively small, so large samples are needed to verify our findings in the future. Second, the current study was a cross‐section research that cannot analyze the dynamic condition of brain iron content in T2DM patients with MCI. Therefore, longitudinal studies are needed to directly assess both brain iron dynamics and cognitive trajectories in T2DM patients with MCI and to explore other potential compensatory processes, such as neuroplasticity or metabolic adaptations. Third, we only used a single method to mearsure brain iron content, which may introduce variance. More methods involved with measuring brain iron load will be applied to enhance our findings.

In conclusion, iron content in the right MTG in T2DM patients with MCI was decreased compared to the T2DM‐NC cases through a voxel‐based comparison. And iron content in the right MTG was negatively associated with memory functions, which may constitute a compensatory mechanism to temporarily delay the progression to dementia for MCI patients. Moreover, GMV of the whole brain and the bilateral MTG were positive moderators on the relationship between MSV of the right MTG and the delayed memory ability. Our findings provided more knowledge about iron metabolism in whole brain and helped us gain a deeper understanding of protective mechanism against dementia in T2DM patients with MCI. QSM may be a helpful MRI technique to probe heterogeneous iron metabolism in CNS for T2DM patients and is worth further exploration.

## Author Contributions

**Qiuyue Zhao:** conceptualization, writing – original draft, writing – review and editing, methodology, software and formal analysis. **Subinuer Maimaitiaili:** investigation, visualization, writing – review and editing. **Yan Bi:** resources, writing – review and editing and supervision. **Ming Li:** resources, writing – review and editing. **Xin Li:** data curation, writing – review and editing. **Qian Li:** data curation, writing – review and editing. **Xinyi Shen:** data curation, writing – review and editing. **Min Wu:** data curation, writing – review and editing. **Linqing Fu:** data curation, writing – review and editing. **Zhengyang Zhu:** resources, writing – review and editing. **Xin Zhang:** resources, writing – review and editing. **Jiu Chen:** resources, writing – review and editing. **Anning Hu:** resources, writing – review and editing. **Zhou Zhang:** data curation, writing – review and editing. **Wen Zhang:** conceptualization, methodology, writing – review and editing, supervision and funding acquisition. **Bing Zhang:** resources, writing – review and editing, supervision, project administration and funding acquisition. **Wen Zhang** and **Bing Zhang** are the guarantors of this work.

## Disclosure

The authors have nothing to report.

## Ethics Statement

The protocol of this study was approved by the Ethics Committee of Nanjing Drum Tower Hospital, in accordance with the Helsinki Declaration and registered at Clinicaltrials.gov (NCT02738671).

## Consent

All participants provided written informed consent after a detailed introduction of the study process according to institutional guidelines.

## Conflicts of Interest

The authors declare no conflicts of interest.

## Supporting information


Data S1.

## Data Availability

The data that has been used is confidential. The datasets generated during and/or analyzed during the current study are not publicly available but are available from the corresponding authors on reasonable request.
