# Supplementary material for: Brain Iron Deposition Alterations in Type 2 Diabetes Mellitus Patients With Mild Cognitive Impairment Based on Quantitative Susceptibility Mapping
Source: J Diabetes. 2025 Jan 22;17(1):e70052. doi: 10.1111/1753-0407.70052 (PMC11753919; doi:10.1111/1753-0407.70052)
Supplement: Supplementary file 1 — Data S1. [file JDB-17-e70052-s001.docx]

**Supplementary table 1-1** | The moderation effects of metabolic factors on the association between MSV of the right MTG and list recognition in T2DM-MCI group

|  | **Effect size** | ***P* value** | **95%CI** |
| --- | --- | --- | --- |
| **HbA1c** |  |  |  |
| MSV of the right MTG | -0.53 | 0.007**^*^** | [-0.91, -0.15] |
| HbA1c | 0.14 | 0.461 | [-0.52, 0.24] |
| Interaction | 0.15 | 0.529 | [-0.32, 0.61] |
| **FBG** |  |  |  |
| MSV of the right MTG | -0.59 | 0.002**^*^** | [-0.96, -0.22] |
| FBG | 0.10 | 0.578 | [-0.27, 0.48] |
| Interaction | 0.00 | 0.988 | [-0.44, 0.45] |
| **PBG** |  |  |  |
| MSV of the right MTG | -0.58 | 0.002**^*^** | [-0.94, -0.22] |
| PBG | -0.20 | 0.295 | [-0.57, 0.18] |
| Interaction | 0.06 | 0.762 | [-0.31, 0.42] |
| **HOMA-IR** |  |  |  |
| MSV of the right MTG | -0.57 | 0.002**^*^** | [-0.93, -0.22] |
| HOMA-IR | 0.34 | 0.075 | [-0.04, 0.71] |
| Interaction | 0.09 | 0.610 | [-0.26, 0.44] |

**Supplementary table 1-2** | The moderation effects of metabolic factors on the association between MSV of the right MTG and list recall in T2DM-MCI group

|  | **Effect size** | ***P* value** | **95%CI** |
| --- | --- | --- | --- |
| **HbA1c** |  |  |  |
| MSV of the right MTG | -0.78 | 0.003**^*^** | [-1.29, -0.28] |
| HbA1c | -0.21 | 0.422 | [-0.71, 0.30] |
| Interaction | -0.03 | 0.926 | [-0.64, 0.59] |
| **FBG** |  |  |  |
| MSV of the right MTG | -0.85 | <0.001**^*^** | [-1.32, -0.37] |
| FBG | 0.43 | 0.082 | [-0.06, 0.92] |
| Interaction | -0.06 | 0.844 | [-0.64, 0.52] |
| **PBG** |  |  |  |
| MSV of the right MTG | -0.82 | 0.001**^*^** | [-1.30, -0.34] |
| PBG | 0.10 | 0.695 | [-0.40, 0.59] |
| Interaction | 0.29 | 0.236 | [-0.19, 0.77] |
| **HOMA-IR** |  |  |  |
| MSV of the right MTG | -0.79 | 0.002**^*^** | [-1.26, -0.31] |
| HOMA-IR | 0.36 | 0.159 | [-0.14, 0.86] |
| Interaction | 0.14 | 0.554 | [-0.33, 0.61] |

**Supplementary table 1-3** | The moderation effects of metabolic factors on the association between MSV of the right MTG and delayed memory in T2DM-MCI group

|  | **Effect size** | ***P* value** | **95%CI** |
| --- | --- | --- | --- |
| **HbA1c** |  |  |  |
| MSV of the right MTG | -5.84 | 0.001**^*^** | [-9.32, -2.36] |
| HbA1c | -0.60 | 0.732 | [-4.11, 2.90] |
| Interaction | -0.16 | 0.940 | [-4.41, 4.09] |
| **FBG** |  |  |  |
| MSV of the right MTG | -6.09 | <0.001**^*^** | [-9.39, -2.78] |
| FBG | 2.46 | 0.149 | [-0.90, 5.82] |
| Interaction | 0.44 | 0.825 | [-3.55, 4.44] |
| **PBG** |  |  |  |
| MSV of the right MTG | -5.99 | <0.001**^*^** | [-9.31, -2.68] |
| PBG | -0.27 | 0.873 | [-3.69, 3.14] |
| Interaction | 1.48 | 0.374 | [-1.82, 4.79] |
| **HOMA-IR** |  |  |  |
| MSV of the right MTG | -5.85 | <0.001**^*^** | [-1.26, -0.31] |
| HOMA-IR | 3.85 | 0.026**^*^** | [0.49, 7.22] |
| Interaction | 0.45 | 0.777 | [-2.72, 3.63] |

Abbreviations: CI, confidence interval; FBG, fasting blood glucose; HbA1c, glycated hemoglobin A1c; HOMA-IR, homeostasis model assessment of insulin resistance; MCI, mild cognitive impairment; MSV, magnetic susceptibility value; MTG, middle temporal gyrus; PBG, postprandial blood glucose; T2DM, type 2 diabetes mellitus.

^*^ *P* < 0.05 was considered significant.

**Supplementary table 2-1** | The moderation effects of cerebrovascular factors on the association between MSV of the right MTG and list recognition in T2DM-MCI group

|  | **Effect size** | ***P* value** | **95%CI** |
| --- | --- | --- | --- |
| **JVWMH volume** |  |  |  |
| MSV of the right MTG | -0.56 | 0.003^*^ | [-0.92, -0.19] |
| JVWMH | -0.07 | 0.773 | [-0.53, 0.40] |
| Interaction | 0.25 | 0.335 | [-0.53, 0.40] |
| **PWMH volume** |  |  |  |
| MSV of the right MTG | -0.58 | 0.002^*^ | [-0.94, -0.22] |
| PWMH | -0.09 | 0.710 | [-0.57, 0.39] |
| Interaction | 0.21 | 0.330 | [-0.21, 0.63] |
| **JCWMH volume** |  |  |  |
| MSV of the right MTG | -0.57 | 0.003^*^ | [-0.94, -0.21] |
| JCWMH | -0.03 | 0.900 | [-0.47, 0.42] |
| Interaction | 0.13 | 0.646 | [-0.42, 0.68] |
| **DWMH volume** |  |  |  |
| MSV of the right MTG | -0.64 | 0.003**^*^** | [-1.06, -0.22] |
| DWMH | 0.22 | 0.503 | [-0.44, 0.89] |
| Interaction | -0.32 | 0.550 | [-1.38, 0.74] |

**Supplementary table 2-2** | The moderation effects of cerebrovascular factors on the association between MSV of the right MTG and list recall in T2DM-MCI group

|  | **Effect size** | ***P* value** | **95%CI** |
| --- | --- | --- | --- |
| **JVWMH volume** |  |  |  |
| MSV of the right MTG | -0.83 | 0.001^*^ | [-1.32, -0.35] |
| JVWMH | 0.15 | 0.631 | [-0.47, 0.76] |
| Interaction | -0.41 | 0.240 | [-1.10, 0.28] |
| **PWMH volume** |  |  |  |
| MSV of the right MTG | -0.78 | 0.002**^*^** | [-1.26, -0.30] |
| PWMH | 0.05 | 0.872 | [-0.57, 0.68] |
| Interaction | -0.38 | 0.177 | [-0.93, 0.17] |
| **JCWMH volume** |  |  |  |
| MSV of the right MTG | -0.80 | 0.001^*^ | [-1.28, -0.32] |
| JCWMH | -0.02 | 0.935 | [-0.61, 0.56] |
| Interaction | -0.35 | 0.337 | [-1.07, 0.37] |
| **DWMH volume** |  |  |  |
| MSV of the right MTG | -0.99 | <0.001**^*^** | [-1.54, -0.45] |
| DWMH | 0.43 | 0.503 | [-0.44, 1.30] |
| Interaction | -1.04 | 0.550 | [-2.43, 0.35] |

**Supplementary table 2-3** | The moderation effects of cerebrovascular factors on the association between MSV of the right MTG and delayed memory in T2DM-MCI group

|  | **Effect size** | ***P* value** | **95%CI** |
| --- | --- | --- | --- |
| **JVWMH volume** |  |  |  |
| MSV of the right MTG | -5.67 | 0.001**^*^** | [-9.00, -2.35] |
| JVWMH | -2.34 | 0.271 | [-6.56, 1.87] |
| Interaction | 0.92 | 0.699 | [-3.81, 5.66] |
| **PWMH volume** |  |  |  |
| MSV of the right MTG | -5.70 | 0.001**^*^** | [-9.02, -2.39] |
| PWMH | -1.96 | 0.370 | [-6.30, 2.38] |
| Interaction | 0.42 | 0.828 | [-3.41, 4.24] |
| **JCWMH volume** |  |  |  |
| MSV of the right MTG | -5.79 | <0.001**^*^** | [-9.09, -2.49] |
| JCWMH | -1.90 | 0.350 | [-5.93, 2.13] |
| Interaction | 0.54 | 0.830 | [-4.44, 5.52] |
| **DWMH volume** |  |  |  |
| MSV of the right MTG | -7.00 | <0.001**^*^** | [-10.76, -3.24] |
| DWMH | 2.36 | 0.44 | [-3.66, 8.37] |
| Interaction | -6.05 | 0.21 | [-15.63, 3.54] |

Abbreviations: CI, confidence interval; DWMH, deep white matter hyperintensity; JCWMH, juxtacortical white matter hyperintensity; JVWMH, juxtaventricular white matter hyperintensity; MCI, mild cognitive impairment; MSV, magnetic susceptibility value; MTG, middle temporal gyrus; PWMH, periventricular white matter hyperintensity; T2DM, type 2 diabetes mellitus.

^*^ *P* < 0.05 was considered significant.
